# Prevalence and influencing factors of oral frailty in older adults: a systematic review and meta-analysis

**DOI:** 10.3389/fpubh.2024.1457187

**Published:** 2024-12-13

**Authors:** Yutong Zhou, Li Zhou, Wen Zhang, Yao Chen, Keyi She, Hongtao Zhang, Yue Gao, Xinhong Yin

**Affiliations:** ^1^School of Nursing, University of South China, Hengyang, China; ^2^Department of Hepatobiliary Surgery, The Second Affiliated Hospital of University of South China, Hengyang, China

**Keywords:** oral frailty, older adults, prevalence, influencing factors, meta-analysis

## Abstract

**Objective:**

This study aimed to conduct a systematic review and meta-analysis, assessing the pooled prevalence and influencing factors of oral frailty in older people to assist healthcare professionals in enhancing their understanding of this condition and formulating efficient interventions.

**Methods:**

This systematic review was performed based on the Preferred Reporting Items for Systematic Reviews and Meta-analyses Statement (PRISMA) guidelines. We searched PubMed, Web of Science, The Cochrane Library, Embase, CINAHL, ProQuest, the National Knowledge Infrastructure (CNKI), WAN FANG DATA, VIP Information, SinoMed and Scopus for literature published in English or Chinese from inception to June 19, 2024. Subsequently, we evaluated the potential for bias in the cross-sectional studies that met our criteria through the Agency for Healthcare Research and Quality (AHRQ) tool. In contrast, we utilized the robust Newcastle-Ottawa scale for cohort and case–control studies. We performed statistical analyses using STATA 17 software. Prevalence was studied using a meta-analysis of a single proportion. For influencing factors, dichotomous variables were expressed as odds ratios (ORs) and 95% confidence intervals (Cls), and continuous variables were expressed as weighted mean differences (WMDs) and 95% confidence intervals (Cls).

**Results:**

Investigation into 17 studies encompassing 24,983 participants discovered a striking 28% overall prevalence rate for oral frailty among older individuals (95% CI 20–36%, *p* < 0.001). Upon assessing the literature’s quality, nine articles acquired high ratings; all others received medium ratings. These findings imply a complex interplay among biological, socio-economic, lifestyle, and disease-pharmacological factors in the manifestation of oral frailty in older adults.

**Conclusion:**

Oral frailty is prevalent in older adults and is impacted by diverse factors. Enhanced surveillance and effective interventions for oral frailty are required in older cohorts.

**Systematic review registration:**

https://www.crd.york.ac.uk/prospero/display_record.php?ID=CRD42024544552, identifier CRD42024544552.

## 1 Introduction

The progression of population aging is escalating globally, thrusting the issue of the level of life in older adults into prominence, with frailty implicated with aging emerging as a crucial area of investigation. Frailty, characterized by a decline in physiological reserves and resilience, is correlated to a myriad of adverse health consequences in older people (1). Oral health issues are also becoming more prominent in older cohorts, and oral health has gradually been recognized as an essential component of overall health. International studies have corroborated the bidirectional relationship between oral health and frailty. Deteriorated oral health in older demographics escalated the risk of frailty, and the manifestation of frailty can further encroach upon oral health status (2). In 2013, oral frailty emerged as a novel concept within the realm of frailty. Oral frailty is a series of phenomena and processes that occur with age when the number of teeth decreases, and oral function and hygiene deteriorate; in older people, it manifests itself as a decrease in attention to oral health, culminating in dysfunctional eating and a decline in physical and mental functioning (3, 4). The most frequently utilized tools for assessing oral frailty are the Oral Frailty Index-6 (OFI-6) (3) and the Oral Frailty Index-8 (OFI-8) (5) developed by Tanaka. Additionally, there is the Oral Frailty Five-item Checklist (6) and the Eating Assessment Questionnaire-10 (7). These tools consider multiple dimensions, including the number of teeth, chewing ability, oral hygiene status, tongue function, pharyngeal function, and nutritional status. We can better understand the intricate relationship between oral frailty and overall health status through comprehensive assessments.

A comprehensive evaluation of numerous domestic and international studies revealed that oral frailty is prevalent within the geriatric populace, exhibiting an overall prevalence of 14.2–44.7% (8, 9). These statistics accentuated the significance of oral frailty as a public health concern, and they mirrored disparities in the oral frailty of older individuals across different regions, socioeconomic backgrounds, and health statuses.

The study of oral frailty in older people revealed that it is not merely a consequence of tooth loss but a complex, multifactorial process involving nutritional intake, social interaction, psychological health, and overall quality of life. Previous research indicated numerous factors contribute to oral frailty. Tanaka proposed that oral frailty is significantly more prevalent within the geriatric demographic (3), possibly because older people are more susceptible to disease. Individuals suffering from cognitive impairment (10), chronic disease (11), and depression (12) are at a higher risk for developing oral frailty. These diseases may indirectly affect oral health through alterations in saliva production, medication side effects, or the physiological impacts of the diseases themselves. Consequently, evaluating and managing oral frailty requires a multidisciplinary approach involving collaboration among nutritionists, dentists, nurses, and other health professionals. It is worth noting that oral frailty in older people can also lead to adverse outcomes, and it not only affects the physical health of older people but also has a profound impact on their social participation and psychological well-being. For instance, falls are a common and serious problem in older adults, and not only can they lead to fractures and other physical injuries, but they can also cause fear and self-limitation, which can reduce social activity and independence (13). Reduced mineral intake can affect bone health and muscle function, which in turn increases the risk of fractures and osteoporosis (14). Muscle atrophy reduces older adults’ mobility and can lead to malnutrition and weakened immunity (15). The decline in quality of life relates to the overall well-being of older people, including physical, mental, and social well-being (16). Addressing oral frailty among older people is a crucial research area warranting our attention.

The discernment and assessment of oral frailty within a reasonable timeframe and understanding its influential factors among older individuals are crucial to mitigating the detrimental impacts of oral frailty. Nevertheless, the prevailing status of this demographic’s combined prevalence and pertinent variables of oral frailty still needs to be clarified. The objective of this systematic review and meta-analysis is to assess the collective prevalence of oral frailty in older people and identify the associated influencing factors, aiming to furnish evidence for healthcare professionals to comprehend oral frailty’s status and devise effective strategies for older individuals, ultimately augmenting the overall health and quality of life for older adults.

## 2 Methods

This systematic review was executed in alignment with the Preferred Reporting-Items for Systematic Reviews and Meta-Analysis (PRISMA) statement (17) and was registered with PROSPERO prior to the initiation of the preliminary exploration (registration No: CRD42024544552).

### 2.1 Search strategy

We searched PubMed, Web of Science, The Cochrane Library, Embase, CINAHL, ProQuest, the National Knowledge Infrastructure (CNKI), WAN FANG DATA, VIP Information, and SinoMed for literature published in English or Chinese from inception to June 19, 2024. Search terms consist of a combination of Medical Subject Headings (MeSH) and free words. The keywords were: (aged OR “older people” OR “older adult*” OR “older individuals”) AND (“oral frailty” OR “oral frail”) AND (impact* OR cause* OR reason* OR association OR relationship OR effect*). The detailed search strategy is shown in Supplementary Table S1 (Pubmed is used as an example). To avoid missing any relevant studies, we also examined all references from the included literature and other appropriate articles.

### 2.2 Inclusion and exclusion criteria

The inclusion criteria were as follows: (a) participants aged 60 years or above with no dementia and no acute infectious diseases; (b) the nature of the study was cross-sectional investigations, cohort studies, and case–control studies; (c) there are explicit diagnostic criteria for oral frailty in the literature; (d) the original literature utilized multifactorial logistic regression analyses to ascertain the risk factors for oral frailty in older individuals. The exclusion criteria were as follows: (a) studies that could not secure full text or failed to extract data; (b) duplicate publications; (c) abstracts, reviews, comments, or conferences; (d) literature with a quality rating of “low.”

### 2.3 Study selection and data extraction

The titles, authors, and abstracts of all the articles procured by the search methodology were imported into EndNote X9 software to eliminate duplicates. Two evaluators independently performed the literature examination and data extraction, verifying the outcomes and referring any discord to a third reviewer for resolution. Ultimately, the literature that met the inclusion criteria was perused in its entirety, and general information (e.g., first author, date of publication, location, participant characteristics) and study characteristics (e.g., sample size, assessment tool, disease prevalence) were summarized in an Excel sheet.

### 2.4 Quality assessment

The caliber of the incorporated original studies was scrutinized, and studies of subpar quality were dismissed to ascertain the credibility of the review findings. The quality assessment of the incorporated studies was conducted independently by two evaluators. Quality assessment of cohort and case–control studies employing the Newcastle Ottawa Scale (NOS) (18). The NOS encompasses eight categories of methodological rigor, and each study was awarded an ultimate score out of a maximum of 9 points, with scores of 0–3, 4–6, and 7–9, denoting low, moderate, and high quality in that sequence. The cross-sectional study was appraised utilizing the Agency for Healthcare Research and Quality (AHRQ) Evaluation Criteria (19), which encompass 11 categories of methodological quality, with each study assigned a score of up to 11 points, with 0–3, 4–7, and 8–11 signifying low, moderate and high quality, respectively. In case of a discrepancy, reconcile it through consensus or consulting a third reviewer.

### 2.5 Statistical analysis

In this research, StataCorp Stata 17 software was employed for statistical evaluation. The prevalence of oral frailty in older adults was studied using a meta-analysis of a single proportion. Weighted mean differences (WMDs) were computed alongside 95% confidence intervals (CIs) to assess influential factors for continuous variables. For dichotomous variables, odds ratios (ORs) and 95% CIs were employed as statistical effect sizes, and a *p*-value < 0.05 signified that the influencing factor was statistically significant. The heterogeneity of the incorporated studies was scrutinized using the I2 statistic. Heterogeneity test *I*^2^ ≤ 50%, indicating less heterogeneity, utilizing a fixed effects model for amalgamation. If *I*^2^ > 50%, heterogeneity was deemed substantial, and a random effects model was implemented. Sensitivity analysis by contrasting the consistency of the computations of the two effects models. Lastly, Egger’s and Begg’s tests evaluated publication bias.

## 3 Results

### 3.1 Search results

**Figure 1:**
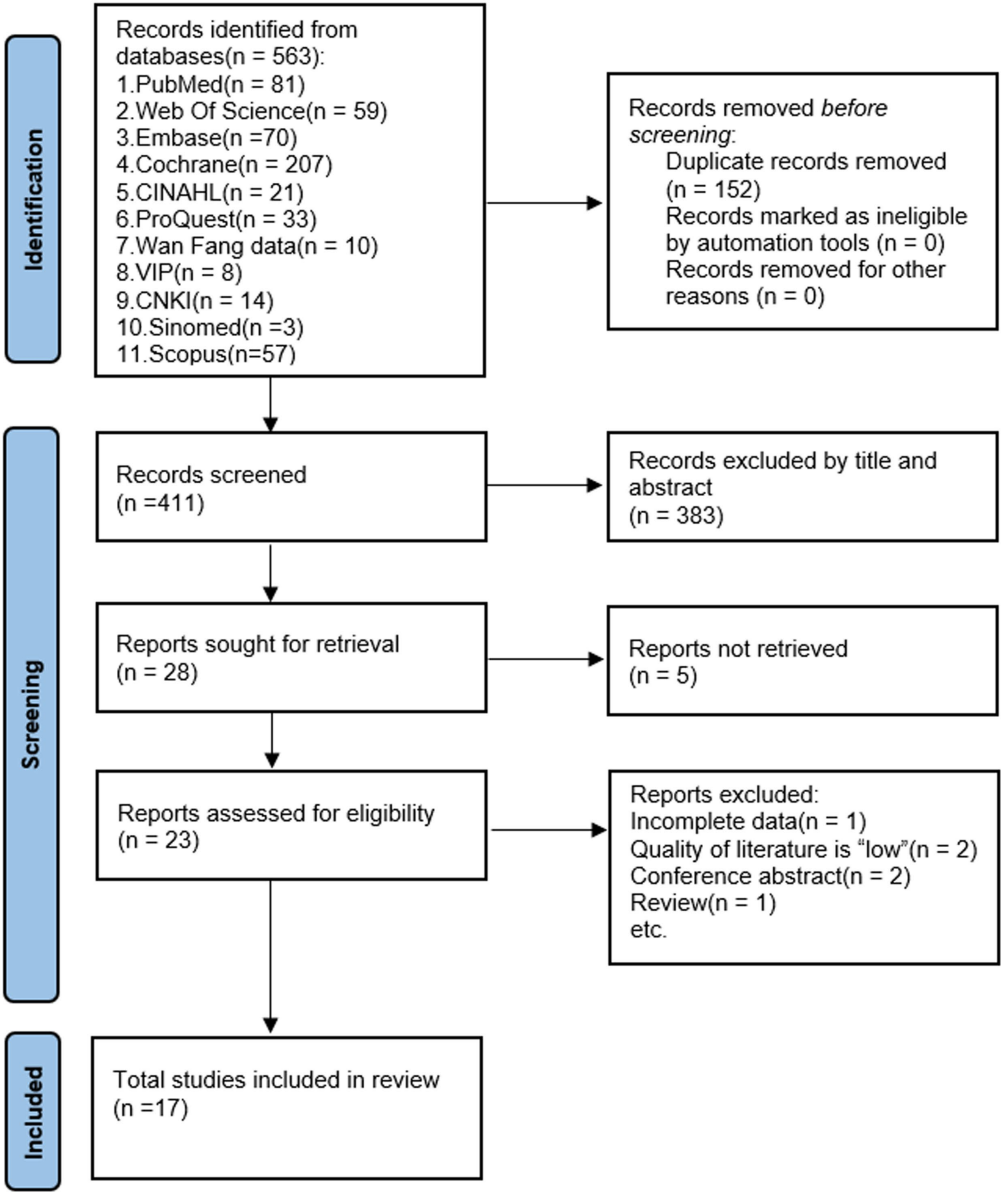
Flow diagram of literature selection.

Figure 1 displays the screening methodology for studies complying with PRISMA regulations. Primarily, utilizing an extensive search method, 563 pertinent references were identified. Duplicates of 152 were eliminated through EndNote X9, targeting paper attributes like title, author, and publication year. Afterward, 411 publications were retained. Following the perusal of abstracts, 383 papers that did not align with the study’s nature or population were discarded. A total of 28 papers were deemed eligible for inclusion. However, we used Find@UNC and interlibrary loan to request articles, of which 23 papers were available in full text. After further reading the complete text, 6 articles that did not satisfy the criteria were excluded, and 17 were included in the meta-analysis.

### 3.2 Characteristics of the included studies

Table 1 illustrates the attributes of all incorporated studies. Regarding the subject features, research was conducted in three diverse nations: nine studies were executed in Japan, five in China, and one in Finland. Thirteen of these studies were conducted in the community, one in a hospital, and one in a nursing care institution.

**Table 1:** Characteristics of included studies.

Author and year of publication	Survey area	Source of research subjects	Number of patients (cases)	Age (years)	Survey tools	Number of oral frailty (cases)	Study design	Quality assessment
Fei et al. (44)	Nanjing, China	Community	307	≥60	OFI-8	65	Cross-sectional study	Moderate
Wu et al. (31)	Anhui, China	Hospital	388	≥60	OFI-8	118	Cross-sectional study	Moderate
Tang et al. (9)	Guizhou, China	Community	1,298	≥60	OFI-8	580	Cross-sectional study	High
Wang et al. (32)	Luzhou, Sichuan, China	Community	223	≥60	OFI-8	132	Cross-sectional study	Moderate
Tu et al. (24)	Hangzhou, China	Community	204	≥60	OFI-8	69	Cross-sectional study	Moderate
Watanabe et al. (45)	Kyoto Prefecture, Japan	Community	11,374	≥65	OFI-8	5,335	Cross-sectional study	High
Izutsu et al. (46)	Sapporo Hokkaido, Japan	Community	238	≥65	OFI-8	24	Cross-sectional study	High
Tanaka et al. (47)	Kashiwa, Japan	Community	2,031	≥65	Oral frailty five-item	799	Cohort study	High
Jiao et al. (33)	Hainan, China	Nursing care institutions	270	≥65	OFI-8	68	Cross-sectional study	Moderate
Misa et al. (48)	Kashiwa, Japan	Community	1,234	≥65	OFI-8	285	Cohort study	Moderate
Tanaka et al. (5)	Kashiwa, Japan	Community	2,011	≥65	OFI-8	319	Cross-sectional study	High
Hiltunen et al. (49)	Helsinki, Finland	Community	349	≥65	OFI-6	62	Cross-sectional study	Moderate
Komatsu et al. (8)	Sasayama-Tamba, Hyogo, Japan	Community	380	≥65	OFI-6	54	Cross-sectional study	Moderate
Hironaka et al. (11)	Tokyo, Japan	Community	682	≥65	OFI-6	65	Cross-sectional study	High
Iwasaki et al. (10)	Tokyo, Japan	Community	1,082	≥70	OFI-6	227	Cross-sectional study	High
Ohara et al. (30)	Tokyo, Japan	Community	722	≥70	OFI-6	139	Cross-sectional study	High
Arai et al. (50)	Tottori Prefecture, Japan	Community	2,190	≥75	OFI-6	972	Cross-sectional study	High

OFI-6, Oral Frailty Index-6; OFI-8, Oral Frailty Index-8.

The sample size encompassed between 204 and 11,374. The total sample size implicated in this research was 24,983 cases, with a cumulative oral frailty sample size of 9,313 cases. In terms of evaluation instruments to delineate oral frailty, 10 studies utilized the Oral Frailty Index-8 (OFI-8), six studies used the Oral Frailty Index-6 (OFI-6), and one used the Oral frailty five-item to ascertain whether the study participants were afflicted with oral frailty.

### 3.3 Quality assessment of included studies

In the study that explored the prevalence of oral frailty in older adults and its influencing factors, we synthesized multiple studies, including 15 cross-sectional studies and 2 cohort study. These studies cover data from different regions, populations, and time points, providing a broad perspective on the oral frailty of older adults. After carefully assessing the quality of these studies, we found that nine studies met the criteria for high-quality studies with good design, appropriate sample sizes, reasonable methods of statistical analysis, and reliable results. Although limited in some areas, the remaining studies provided valuable information and were classified as moderate quality. The results of these studies are critical for understanding the epidemiological profile of oral frailty in older adults (Supplementary Tables S2, S3).

### 3.4 Pooled prevalence of oral frailty in older adults

**Figure 2:**
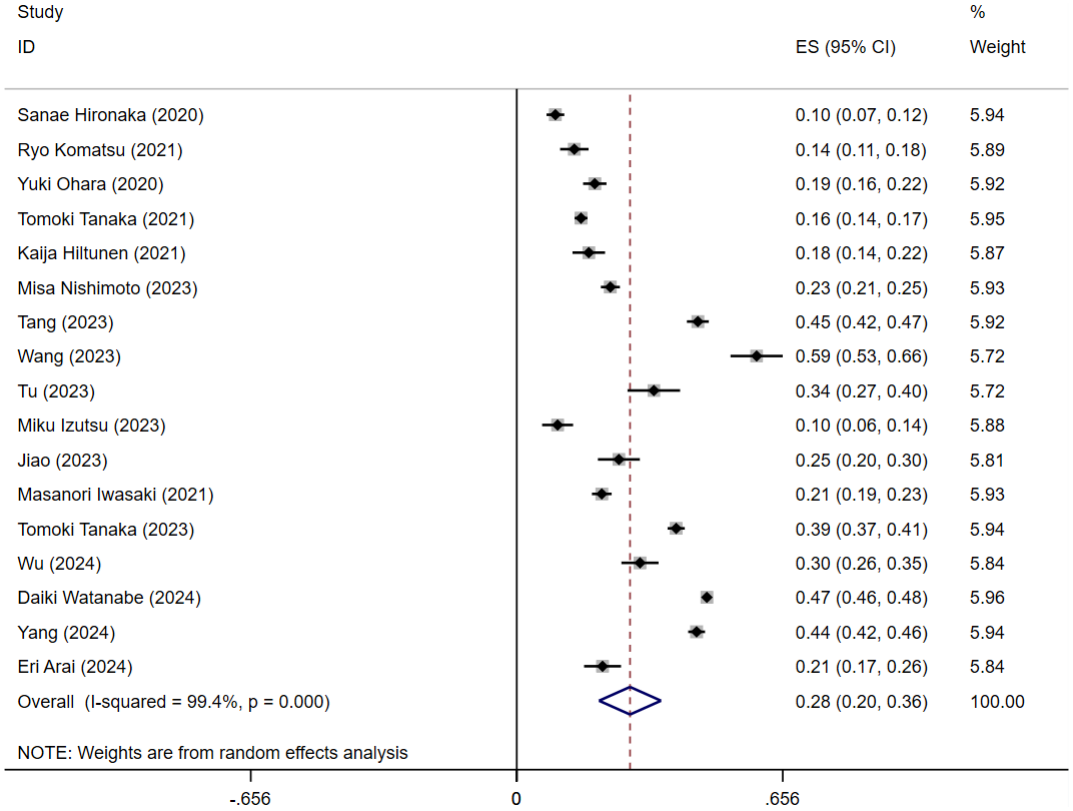
Forest plot of the pooled prevalence of oral frailty in older adults.

A comprehensive meta-analysis was conducted, including 17 studies comprising a total of 24,983 older adults, to investigate the prevalence of oral frailty in geriatric populations. The prevalence of oral frailty varied widely across the 17 studies, ranging from 9.53 to 59.19%. Figure 2 illustrates the findings of the random-effects model, which revealed a pooled prevalence of oral frailty in older adults at 28% (95% CI 20–36%). However, it is essential to note the significant heterogeneity across the studies (*I*^2^ = 99.4%, *p* < 0.001).

### 3.5 Subgroup analysis

The results of the subgroup analysis of the prevalence of oral frailty are presented in Supplementary Table S4. Notably, studies conducted in China reported a higher prevalence of oral frailty (40, 95% CI 32–47%) than those from Japan (22, 95% CI 11–33%). When different assessment tools for oral frailty were used, the prevalence varied, with studies using OFI-6 and OFI-8 to define oral frailty reporting a prevalence of 21% (95% CI 14–28%) and 31% (95% CI 21–41%) respectively. Pooled prevalence was similar in subgroups with different study designs.

### 3.6 Systematic review and meta-analysis of factors influencing oral frailty in older people

After integrating the results of the original study, we identified a total of 22 influencing factors. We classified them into four main categories: biological factors, lifestyle factors, socio-economic factors, and disease-drug-related factors. In this study, we identified 22 distinct influencing factors. Of these, 20 were dichotomous variables. For these dichotomous variables, we employed a meta-analytical approach designed explicitly for such variables, with the results presented in Table 2. The remaining two factors were continuous variables. Therefore, we utilized a meta-analysis method appropriate for continuous variables. This methodological distinction ensures the accuracy and reliability of our analysis. There were two continuous variables, age, and body mass index (BMI), and the forest plots were shown in Supplementary material. Finally, the pooled estimates showed that age (WMD = 2.84, 95% CI 2.26–3.43, *p* = 0.002); women (OR = 1.20, 95% CI 1.05–1.37, *p* = 0.008); difficulty in chewing (OR = 13.70, 95% CI 10.33–18.17, *p* < 0.001); dysphagia (OR = 3.93, 95% CI 2.26–6.83, *p* < 0.001); nutritional disorders (OR = 2.95, 95% CI 2.18–3.99, *p* < 0.001); physical frailty (OR = 2.16, 95% CI 1.16–2.90, *p* < 0.001); spousal-absence (OR = 1.80, 95% CI 1.03–3.17, *p* = 0.041); low income (OR = 1.57, 95% CI 1.29–1.91, *p* < 0.001); taking≥5 medications (OR = 3.30, 95% CI 1.15–9.47, *p* = 0.026); stroke (OR = 1.62, 95% CI 1.13–2.33, *p* = 0.009); urological disease (OR = 1.38, 95% CI 1.17–1.63, *p* < 0.001); diabetes (OR = 1.17, 95% CI 1.06–1.29, *p* = 0.001); hypertension (OR = 1.31, 95% CI 1.17–1.47, *p* < 0.001); heart disease (OR = 1.41, 95% CI 1.09–1.82, *p* = 0.010); osteoporosis (OR = 1.50, 95% CI 1.28–1.77, *p* < 0.001); depression (OR = 2.12, 95% CI 1.61–2.79, *p* < 0.001) were risk factors of oral frailty, forest diagram as shown in Supplementary material. However, the pooled data indicated no significant difference in the association between oral frailty and BMI, drinking, residing solitary, malignant neoplasm, and dyslipidemia. Due to differences in the primary data, two influencing factors (low literacy level and abnormal dietary structure) could not be included in the meta-analysis, so we conducted a systematic review. In the end, it can be concluded that both factors may contribute to oral frailty in older people.

**Table 2:** The outcomes of the meta-analysis of dichotomous variable.

Potential factors	Number of studies	Heterogeneity test		Effect model	Meta-analysis results		Egger’s test (*p*)	Begg’s test (*p*)
		*I*^2^ (%)	*p*		*p*	OR (95%CI)		
**Biological factors**								
Women	17	69.2	<0.001	Random effects model	0.008	1.20 (1.05–1.37)	0.264	0.902
Difficulty in chewing	4	0.0	0.891	Fixed effects model	<0.001	13.70 (10.33–18.17)	0.320	0.734
Dysphagia	4	77.9	0.004	Random effects model	<0.001	3.93 (2.26–6.83)	0.312	0.308
Nutritional disorders	6	0.0	0.446	Fixed effects model	<0.001	2.95 (2.18–3.99)	0.900	0.707
Physical frailty	5	41.3	0.146	Fixed effects model	<0.001	2.16 (1.16–2.90)	0.845	0.806
**Lifestyle factors**								
Preexisting or former smoker	10	66.0	0.002	Random effects model	0.112	1.21 (0.96–1.53)	0.092	0.283
Presently or prior alcohol consumption	10	74.8	<0.001	Random effects model	0.158	0.86 (0.71–1.06)	0.376	0.107
Residing solitary	7	63.0	0.013	Random effects model	0.125	1.19 (0.95–1.50)	0.130	0.764
**Socio-economic factors**								
Spousal-absence	5	85.7	0.001	Random effects model	0.041	1.80 (1.03–3.17)	0.440	0.221
Low income	9	66.2	0.003	Random effects model	<0.001	1.57 (1.29–1.91)	0.172	0.466
**Disease-pharmacological factors**								
Malignant neoplasm	7	93.1	<0.001	Random effects model	0.178	1.45 (0.85–2.47)	0.234	0.764
Taking ≥5 medications	4	83.6	<0.001	Random effects model	0.026	3.30 (1.15–9.47)	0.963	1.000
Stroke	6	58.8	0.033	Random effects model	0.009	1.62 (1.13–2.33)	0.708	1.000
Urological diseases	6	0.0	0.589	Fixed effects model	<0.001	1.38 (1.17–1.63)	0.231	0.260
Diabetes mellitus	10	37.8	0.107	Fixed effects model	0.001	1.17 (1.06–1.29)	0.553	0.474
Hypertension	8	60.7	0.013	Random effects model	0.019	1.20 (1.03–1.47)	0.408	0.536
Heart disease	9	45.9	0.063	Fixed effects model	<0.001	1.42 (1.29–1.56)	0.782	0.754
Dyslipidemia	7	73.9	0.001	Random effects model	0.372	0.91 (0.74–1.12)	0.386	0.764
Osteoporosis	7	0.0	0.922	Fixed effects model	<0.001	1.50 (1.28–1.77)	0.020	0.072
Depression	3	8.3	0.336	Fixed effects model	<0.001	2.12 (1.61–2.79)	0.966	1.000

### 3.7 Sensitivity analysis and publication of bias

Sensitivity analysis is a statistical method used to determine whether changes in certain variables or data sets will significantly impact a study’s results. In this study, the application of sensitivity analysis confirmed the high reliability of our meta-analysis results, and the results of combined prevalence and influencing factors remained stable even under different statistical models or study selection criteria.

Publication bias refers to the selectivity of literature publication due to the nature of the findings, which may affect the accuracy of the findings. Egger’s and Begg’s tests are two standard statistical methods used to detect publication bias in meta-analysis. In this study, these two tests were selected to assess the possibility of publication bias. In our study, neither of these tests showed significant publication bias, thus increasing confidence in the results.

In conclusion, our findings suggest that the combined prevalence of oral frailty and its influencing factors in older adults are statistically stable and reliable. They are not significantly affected by publication bias during the study. This provides a solid basis for further research on oral vulnerability in older adults and a scientific basis for developing relevant prevention and interventions.

## 4 Discussion

### 4.1 Prevalence of oral frailty in older adults

The meta-analysis incorporating 17 original studies revealed the prevalence of oral frailty in older individuals to be 28%. Substantial heterogeneity in the result was noted, potentially rooted in the instrumentation employed in the study, the region under scrutiny and study designs. Consequently, subgroup analyses were conducted based on these three facets. Nevertheless, substantial heterogeneity persisted in post-subgroup analyses, prompting us to opt for a random-effects model to amalgamate the prevalence ultimately.

Within the Subgroup analysis constructed around the diagnostic tool, we discerned a heightened assessment rate of oral frailty on the OFI-8 compared to the OFI-6, a discrepancy potentially derived from disparities in entry construction and content scope between the two scales.

Six of the 17 studies included in this study applied the OFI-6, and the prevalence of oral frailty derived from integrating the studies’ results was 21%. Ryo Komatsu et al. revealed that the OFI-6 is meticulously and scientifically executed, and its validity is predicated on the precision of its professionals. These professionals not only undergo rigorous training but are also outfitted with cutting-edge dental apparatus to ensure the accuracy and dependability of the assessment procedure (8). During the evaluation process, the professionals will numerically quantify four pivotal objective indicators, which encompass the number of teeth—a fundamental indicator that directly mirrors the state of oral health; masticatory competence—appraises whether the individual can effectively grind food into minute particles suitable for ingestion; oral articulate function—reflecting the harmony of oral structures and muscle motions; and tongue compression—a metric of the tongue’s capacity to generate pressure in the oral cavity, which is intimately associated with swallowing and articulate function (3). The quantitative measurement of these objective indicators provides a solid scientific basis for assessment. In addition, the OFI-6 incorporates the patient’s subjective perception report on feeding and swallowing difficulties. This combination of subjective and objective assessment modes makes the assessment results more comprehensive and detailed. It focuses on the physiological aspects of oral function and considers the patient’s life experience and self-perception, thus providing a multidimensional assessment perspective. Despite the pivotal role of the OFI-6 in assessing oral frailty, its specialized nature, coupled with stringent requirements for specialized equipment and expertise, limits its broad utilization in swift screening or comprehensive community studies. This constraint has fueled the pursuit of more adaptable and user-friendly assessment instruments to satisfy the assessment demands in various contexts.

The OFI-8 stands out for its flexibility and ease of administration. The scale consists of 8 self-assessment items covering several dimensions related to oral frailty, such as “more difficult to eat hard food compared to 6 months ago,” “choking when drinking tea or soup recently,” “use of denture,” “often feel dry in the mouth,” “able to chew hard food,” and “often feel dryness in the mouth,” “do you brush your teeth at least 2 times a day” and “do you visit the dentist at least once a year” (5). The OFI-8 was meticulously engineered to provide a more thorough evaluation by encompassing oral health-related behaviors, lifestyle customs, and social involvement while preserving certain dental specialty examination elements. Investigations utilizing the OFI-8 instrument demonstrated an escalated prevalence of oral frailty in older individuals to 31%. This elevation not only portrays the merits of the OFI-8 scale in terms of diagnostic efficacy but also signifies that a comprehensive analysis of the ramifications of multidimensional factors is imperative when examining oral frailty (20). This multifaceted assessment methodology not only elevates the precision of discerning oral frailty and efficiently discerns potential instances of oral frailty but also diminishes the threshold of evaluation, enabling the process to be accomplished without the direct involvement of a dental professional, expediting its application on a broader scale. The superior sensitivity of the OFI-8 renders it a productive instrument for ascertaining the risk of oral frailty. Hence, the OFI-8 scale not only provides a practical and potent method for the general investigator to monitor and scrutinize the onset and progression of oral frailty in older individuals but also, through its implementation, we can attain a more profound comprehension of the intricacy of oral frailty, subsequently, devise more precise preventive and therapeutic strategies to enhance the oral health and standard of living of older people.

In summary, the OFI-8 and OFI-6 possess unique advantages, and scale selection should hinge on a blend of specific research objectives, resource constraints, and the attributes of the target population. Future research ought to concentrate on creating more comprehensive oral frailty assessment instruments.

Moreover, in our subgroup analysis centered around the country, we discerned a heightened prevalence of oral impairment in Chinese older people, which contrasted with Japan, potentially reflecting disparities in the oral healthcare system methodology. In Japan, oral health dilemmas for older individuals have been assimilated into national policies, mainly through universal dental screening, emphasizing comprehensive oral health care methods. Concurrently, the Japanese healthcare system grants suitable dental care services for older adults, highlighting the importance of averting oral diseases (21). Japan persists in endorsing and fortifying existing oral health policies, mainly the function of in-hospital dentistry for oral diagnosis and treatment of older people and the significance of domestic dental care, and further amalgamate medical and dental services to offer more convenient oral health management for older adults. In China, the focus on the oral health of the geriatric populace has recently augmented substantially, underscoring the correlation between oral health and overall wellness via the Healthy Mouth Initiative (2019–2025) (22). Nevertheless, several establishing challenges exist in executing this policy, such as propagating oral health understanding more efficiently, particularly in rural and underserved regions, and enhancing equal accessibility to dental services for diverse socioeconomic entities (23). We advocate reinforcing public education initiatives and augmenting oral health literacy. The nation should invest substantially in training and deploying dental health professionals to underserved areas. Develop community-based initiatives to endorse preventive oral health practices.

Furthermore, we contend that digital transformation is becoming pivotal to enhancing the frequency and quality of dental health services for older adults in present healthcare models (22). Healthcare practitioners can leverage digital platforms and telemedicine to broaden the spectrum of dental health education and services. Primarily, by establishing electronic medical records, we can attain incessant recording and information sharing of residents’ dental health data, which not only aids healthcare workers in remotely scrutinizing the dental health status of older individuals but also furnishes personalized health administration recommendations. Secondly, by employing telemedicine technologies such as 5G, we can provide remote consultation services, enabling inhabitants in geographically isolated regions to receive professional dental health services, optimizing the allocation of medical resources, and enhancing the efficacy of medical services. Moreover, via the online platform for dental health education, we can augment the comprehension of dental health and self-management aptitude of older people, and the simulated clinical procedures and experiments provided by the digital instructional platform further enrich the learning experience. Concurrently, forming a multidimensional, multidisciplinary collaborative treatment (MDT) paradigm offers patients a holistic and individualized treatment strategy, which boosts the efficiency of diagnosis and treatment and curtails the consultation time, particularly for patients requiring interdisciplinary collaborative treatment. Lastly, the establishment of a nationwide dental health monitoring network, the regular collection, and examination of information on the prevention and treatment of dental pathologies, the fortification of data analyses and utilization, and the practical assessment of the effectiveness and cost-effectiveness of prevention and treatment methodologies are essential to the consistent improvement and optimization of services.

Through these strategies, we can ensure the sustainability, customization, and efficacy of oral health services for older people and accommodate the varied and personalized oral health requirements of the populace.

### 4.2 Factors influencing oral frailty in older adults

The findings of this research underscored the need for a comprehensive approach to managing oral frailty in older people. This included addressing the biological and socio-economic determinants, managing disease-related and pharmacological factors, and considering the lifestyle aspects that influence oral health behaviors. Such an approach can lead to more effective prevention and management strategies, ultimately improving the quality of life for older adults.

#### 4.2.1 Biological factors

Aging induces several transformations within the oral cavity, potentially leading to numerous pathologies. Reduced alkaline phosphatase activity in periodontal cells is particularly noteworthy as it signifies diminished bone formation and repair capability. This function is integral to preserving the structural integrity of the jaw and dental structure. The reduced regenerative potential of periodontal stem cells further diminishes the natural recuperative processes of the oral cavity, rendering it more vulnerable to chronic oral disorders. In addition, advanced age can lead to the physiological decline of the gums, leading to increased root exposure and an increased risk of root surface caries. This exposure, coupled with demineralization and softening of the alveolar bone, cultivates an environment conducive to the colonization of cancer-inducing bacteria and the progression of dental caries. Collectively, these factors contribute to a weakened periodontal support system, culminating in an elevated risk and prevalence of oral frailty in older people (24). To effectively manage the challenges associated with oral frailty in older people, healthcare professionals must thoroughly comprehend the physiological modifications that occur with aging.

Chewing and swallowing are crucial for preserving oral health, particularly for older people. These functions facilitate the mechanical digestion of food and are the initial phase in the digestive process. With aging, these pivotal oral functions naturally diminish. This decline frequently prompts older individuals to favor softer, more fluid foods (25). Consequently, more challenging solid foods necessitating substantial chewing and swallowing efforts are consumed less. This dietary shift can inadvertently induce an imbalance in nutrient intake. Nutrient deficiencies can result in an insufficient supply of nutrients to the structure of the mouth, moderate gum recession, and a reduction in the number of teeth (26). Difficulty chewing and swallowing mechanism impairment prolongs the residence time of food particles in the oral cavity, thereby augmenting the likelihood of bacterial colonization and instigating periodontitis (27). It has been hypothesized that oropharyngeal dysphagia may induce diminished salivary flow, desiccation of the oral cavity, and a reduction in the capacity of the oral cavity to automatically cleanse, potentially affecting oral health. Geriatric patients should be routinely evaluated by a qualified medical practitioner for oral health, encompassing an evaluation of the number of teeth, mastication function, and oral motor performance. This aids in identifying issues that may lead to debilitating oral conditions. Healthcare professionals can also devise oral exercises, such as mouth-opening routines, tongue pressure resistance drills, and rhythm training.

The correlation between physical and oral frailty constitutes a pivotal area of concern within geriatric health. Physical frailty is defined by a depletion in physiological reserves and a diminution in physical fortitude. This condition engenders decreased social engagement and curtails opportunities for verbal communication, which are indispensable for preserving cognitive function and emotional well-being (11). The suboptimal utilization of oral and maxillofacial muscles, coupled with the tongue’s reduced functional capacity, including a diminished ability to exert pressure with the tongue, compromised mastication efficiency, difficulties in swallowing (deglutition), and a discernible deceleration of tongue movements. These factors collectively contribute to a deterioration in oral health.

In addition to the aforementioned biological factors, our analyses revealed that older adults diagnosed with mild cognitive impairment were more susceptible to oral frailty. As cognitive impairment progresses, older individuals demonstrate reduced capabilities for activities of daily living and encounter difficulties in self-maintenance of oral hygiene. Furthermore, oral frailty scores were notably negatively correlated with orientation, memory, attention and numeracy, recall, and total MMSE scores (28). Notably, the concurrence of physical frailty and mild cognitive impairment is quite prevalent in geriatric populations, and “cognitive frailty” refers to a clinical syndrome in which physical frailty and cognitive impairment coincide (29). Consequently, cognitive frailty can result in difficulties with feeding, swallowing, and oral self-perception in older adults. These impairments may further impede older adults’ nutritional intake and overall health status, amplifying oral frailty’s evolution. Hence, prompt recognition of cognitive frailty in older adults is instrumental in preventing oral frailty.

#### 4.2.2 Socio-economic factors

In recent years, the dyadic coping model has been extensively applied in diverse investigations. The extensive application of the model reveals the importance of mutual reminders and supervision between partners in maintaining oral hygiene habits among older adults. The presence of a spouse can help older adults maintain good oral hygiene through daily interactions and support, thereby reducing the risk of oral diseases. However, spouseless older adults often lack this form of support, which can lead to neglect of oral hygiene and increase the likelihood of developing oral diseases (30). Caregivers ought to underscore the significance of oral health, facilitate daily oral hygiene routines predicated on the requirements of unmarried older adults, select suitable oral care products, and affix regular dental check-ups and treatments while providing care.

The education level of older adults also has a particular impact on oral frailty (24, 31, 32). The prevalence of oral frailty was elevated in the uneducated compared to the educated, presumably due to those with a deficient educational level possessing substandard health literacy, being less inclined to seek oral health care knowledge independently, disregarding the prevention and treatment of oral diseases, and exhibit poor oral hygiene practices such as inadequate tooth brushing duration and incorrect technique, resulting in diminished oral muscle strength, inferior chewing function, bleeding gums, and other issues. Clinical professionals, social operatives, and caregivers should synergize to orchestrate workshops, disseminate literature, and provide individualized consultation.

One of the results in the meta-analysis was congruent with a previous investigation where low income contributed to oral frailty among older adults. Older individuals from low-income backgrounds often lack the economic resources to procure high-quality oral care items. Due to fiscal constraints, they may need more capacity to afford routine oral examinations and treatments, potentially leading to oral disorders not being diagnosed and addressed promptly (3). Furthermore, financial constraints may prompt them to diminish their dietary selections in favor of less costly foods possessing diminished nutritional value, which may negatively influence oral health. For older people with restricted economic resources, healthcare practitioners and social workers should collaborate to devise and disseminate resource materials that delineate the steps to apply for financial aid. Moreover, it is imperative to establish support networks within community health centers and dental facilities that can offer personalized guidance to older people in navigating applications for financial assistance.

#### 4.2.3 Lifestyle habits

Although the combined results of this study on the effect of lifestyle habits on oral frailty in older adults were not statistically significant, the impact of poor lifestyle habits on the oral health of older adults cannot be ignored. It is noteworthy that among the original literature included, six studies clearly showed that smoking is one of the factors contributing to oral frailty in older adults (9–11, 30, 32, 33). As nicotine reduces oral microbial diversity and disturbs the oral microecological equilibrium, it stimulates the proliferation of Streptococcus, consequently exacerbating the progression of dental caries (30). Moreover, smokers are at an increased risk of malignant transformations in oral leukoplakia, elevating the probability of oral leukoplakia evolving into cancer. It is of paramount significance to urge older adults to cease the consumption of tobacco products. The process of ceasing tobacco should be bolstered by a comprehensive strategy encompassing education, counseling, and, when indicated, pharmacotherapy.

Two separate studies have shown that older people who prefer diets high in protein, fat, and salt, especially those who eat a predominantly meat-based diet, are at higher risk of oral frailty. This finding has led to an in-depth discussion of the relationship between eating habits and oral health in older adults. An unreasonable diet, especially excessive intake of spicy, greasy, and salty heavy foods, can lead to an inadequate intake of essential nutrients, especially vitamins and minerals, necessary for maintaining oral health (9, 31). It has been determined that older individuals exhibiting a fondness for sweets appear to be more susceptible to oral health complications. The scientific rationale for this phenomenon lies in the capacity of oral bacteria to catabolize sugar and generate acids that erode tooth enamel, precipitating the onset of dental caries (34). Excessive sugar consumption fosters plaque development; if it is not eliminated promptly, it will progressively calcify to form calculus, further exacerbating the deterioration of oral diseases (35). Older individuals should be encouraged to subsist on a diet fortified with calcium, phosphorus, and vitamin C, which are integral for sound gums, and to refrain from immoderate sugar consumption. Furthermore, foods that stimulate salivary production, such as fiber-rich fruits and vegetables, can aid in naturally cleansing the oral cavity and safeguarding against decay. Healthcare professionals must advocate for nutritional evaluations as part of standard health screenings for older people. These evaluations can detect nutritional deficiencies and culminate in the formulation of personalized dietary plans that accommodate the unique requirements of older individuals.

It is crucial to highlight that fostering sound oral management habits can deter the onset of oral frailty in older individuals. Effective oral hygiene practices can mitigate prevalent oral diseases such as dental caries and periodontal disease, and robust teeth and gums assist with masticating and swallowing, ensuring that older people are capable of consuming adequate nutrition (36). Caregivers play a critical role in enhancing oral health among the geriatric population. Their responsibility to disseminate comprehensive education concerning oral hygiene practices, underscoring the paramount importance of thorough brushing and flossing methodologies. These foundational skills form the cornerstone of oral care and are indispensable in averting dental decay and periodontal diseases, which can result in oral frailty.

Consequently, caregivers should dig deeper into how lifestyle habits affect the oral health of older adults through different pathways. This will help us develop more effective prevention and interventions to improve oral hygiene and the overall quality of life and well-being of older adults. Through these measures, we can provide a healthier and more active living environment for older people, thereby reducing the occurrence of oral frailty.

#### 4.2.4 Disease-pharmacological factors

The interrelation between systemic diseases and pharmacological interventions is critical in developing and progressing oral frailty in older people.

In recent years, an amplified interest in the diagnostic evaluation and therapeutic management of chronic ailments in the geriatric population has been witnessed across numerous nations. Beyond causing a gradual erosion in physical function in older adults, chronic diseases also significantly influence oral health. The outcomes of the meta-analysis indicate that diabetes and hypertension serve as determinants of oral frailty in older individuals. Diabetic individuals often exhibit an augmentation in glucose concentration within saliva. This oral milieu creates optimal conditions for bacterial proliferation, thereby rendering diabetic patients a high-risk cohort for periodontitis. Periodontitis instigates a chronic low-grade inflammatory response, culminating in compromised mitochondrial functionality and elevated oxidative stress. These pathological phenomena potentially lead to muscle mass depletion. When this muscle degradation afflicts the masticatory muscles, oral function will considerably diminish (37). Hypertension may impede the body’s calcium metabolism equilibrium, accelerating calcium depletion. Particularly when the systolic blood pressure ascends or surpasses 148 mmHg, bone mineral density will diminish rapidly (38). This calcium loss not only significantly impacts generalized bone health but may also accumulate in the alveolar bone, jeopardizing the stability of teeth and disrupting the normal function of oral motions. Notably, vascular sclerosis and decreased circulation can impair the blood supply to oral structures, culminating in reduced molars (39). Furthermore, cardiovascular disease can result in a reduction in immunity and oral self-regenerative capacity, rendering older adults more vulnerable to oral bacterial infections and, ultimately, a plethora of oral complications (40).

Within Traditional Chinese Medicine (TCM), the tenet of systemic interconnectedness postulates that the integrity of the urinary system, specifically the kidneys, may have an indirect yet substantial influence on oral health (41). Conforming to TCM precepts, the kidneys are not merely crucial in the urinary system but also possess a profound correlation with the oral cavity via the body’s meridian system. Kidney function is fundamental to the regulation of mineral and bone metabolism. With the weakening of renal function, the prevalence of metabolic bone disease, particularly osteoporosis, is often observed in older people (14). Osteoporotic individuals exhibit an elevated risk of bone deterioration in the jawbone, rendering the periodontal tissues more vulnerable to injury and augmenting the likelihood of periodontitis. Disorders such as loosened and dislodged teeth and periodontitis can induce pain in the oral region. This discomfort may intensify oral discomfort during meal consumption by older individuals, further jeopardizing oral health. For osteoporotic patients, proactive strategies such as calcium and vitamin D supplementation, coupled with medications that fortify bone density, may prove beneficial for their overall skeletal health and mitigating the risk of oral frailty.

Many older adults have multiple concurrent medical conditions and require various medications to manage their conditions. The interactions between the use of numerous drugs and oral health outcomes are intricate in older adults. An abnormal proliferation of gingival tissue characterizes drug-induced gingival hyperplasia. Excess gingival tissue creates the conditions for bacterial colonization, which ultimately leads to persistent inflammation and sets the stage for periodontal disease, which is a severe risk to oral health. These diseases not only cause discomfort but also lead to deterioration of oral function, which manifests as oral frailty. It is recommended that healthcare providers be aware of the mechanisms and side effects of various medications, carefully monitor the oral health status of patients using multiple medications, and include dental evaluations as part of standard care (42).

The mental health of geriatric patients significantly influences their oral health status. Geriatric patients afflicted with depression exhibit heightened chances of oral frailty. In a survey, a heightened prevalence of dysphagia and oral malodor was observed in patients prescribed antidepressants (12). Furthermore, patients experiencing depression exhibit consistent adverse attitudes and health risk behaviors. Mood disorders are associated with immune dysregulation, leading to an increased incidence of pathogenic microbial infections and amplified destruction of periodontal tissues, thereby influencing oral health (43). Positive counseling and prompt intervention can mitigate the influence of mental health issues on oral health.

In conclusion, managing oral frailty presents a formidable challenge, necessitating a comprehensive, long-term strategy from multiple viewpoints. Our meta-analysis consolidates four primary factors influencing oral frailty in older individuals, offering invaluable new insights for subsequent intervention studies. Based on these findings, caregivers can implement the following strategies to facilitate oral frailty in older adults. Initially, we can employ scales to evaluate the oral frailty status of older individuals and gain insight into the factors influencing it by following up or reviewing their medical records. Then, we must enhance older individuals’ comprehension of oral frailty through health education.

Moreover, we urge and guide older individuals to improve their lifestyle habits, such as smoking cessation, alcohol restriction, balanced diet, and regular oral hygiene, essential to prevent oral frailty. Secondly, the collaboration of the healthcare team is also pivotal in closely monitoring the physiological functions of older adults and the progression of the disease, thereby continually optimizing the treatment and care plan and mitigating the factors detrimental to oral health. Simultaneously, community support frameworks are being advanced to provide indispensable oral health care for low-income, low-education older adults, including routine public service assessments and oral frailty counseling services. Collaborative endeavors among dental specialists, dieticians, social workers, and primary care physicians can establish comprehensive healthcare management services for the geriatric population. Moreover, digital health platforms and telemedicine technologies are applied to offer expedient oral health evaluation and monitoring services for older adults, which are pivotal for those residing in remote regions or with restricted mobility. Enhancing the residential environment of older people and providing age-appropriate oral hygiene tools and facilities to facilitate their daily oral care is also a crucial aspect of improving their quality of life. Lastly, implementing a continuous monitoring and feedback mechanism to routinely evaluate the efficacy of oral impairment interventions and modify the care plan based on the feedback will assist us in continually refining our care strategies to accommodate the evolving health requirements of older people.

Through these comprehensive long-term strategies, we shall not only be capable of establishing a healthier and more active living environment for older individuals and diminishing the prevalence of oral frailty but also enhance their overall quality of life and well-being. Execution of these measures necessitates the cooperative efforts of care providers, healthcare teams, community service providers, and policymakers to ensure that the oral health of older individuals receives inclusive and sustained attention. Through this interdisciplinary and multilevel collaboration, the complications of oral frailty in older individuals can be more proficiently addressed to safeguard their oral health.

### 4.3 Strengths and limitations

The meta-analysis was implemented to be exhaustive and provide a quantitative integration of the prevalence and determinants affecting oral frailty in older adults, thereby discerning effects that may be elusive to identify in a single study due to limited sample sizes, yielding more credible results. The methodological component of this research was rigorous, and we assessed the incorporated studies’ quality, including methodological rigor, sample selection, and data integrity. This meticulous evaluation aided in detecting potential study biases and constraints and augmenting the findings’ dependability.

Nevertheless, certain limitations exist within this study. Firstly, although we extensively explored multiple databases, database indexing discrepancies could lead us to pay attention to pertinent studies. Subsequently, the originative studies on older individuals originated from diverse sources, and the influential factors of the studies varied. Furthermore, it was noted that the outcomes attained when assessing the prevalence of oral frailty utilizing different assessment tools exhibited significant variances. Still, we have yet to locate an oral frailty assessment instrument that can be used universally for all older adults. Moreover, studies on oral frailty in older adults have been centered on Japan and China, which are regional and do not encapsulate the current status of oral decline in older individuals globally. Ultimately, most of the original studies incorporated in this study were cross-sectional, restricting the establishment of causal relationships. Consequently, future studies advocate more extensive prospective cohort investigations with stringent designs and broad sample sizes to establish a foundation for subsequent research on oral frailty in older people.

## 5 Conclusion

In summation, this study indicates that the prevalence of oral frailty in older people is 28%. Furthermore, age, no spouse, concurrent administration of ≥5 pharmaceuticals, hypertension, diabetes, heart disease, masticatory difficulties, swallowing impairment, physical frailty, aberrant nutritional status, limited financial resources, and depression are risk factors for oral frailty in older adults. In contrast, appropriate oral hygiene management is a preventative measure against oral frailty. These findings suggest that screening for oral frailty in older adults should be strengthened, the underlying mechanisms of oral frailty should be further explored, and how its impact on the quality of life of older adults can be mitigated through prevention and intervention. In addition, longitudinal studies are necessary to understand the progression of oral frailty over time and the effectiveness of various intervention strategies. Through these efforts, we can expect further progress in oral health in older people, ultimately improving the health of older adults.

## Data Availability

The original contributions presented in the study are included in the article/Supplementary material, further inquiries can be directed to the corresponding author.
