# Supplementary material for: Prevalence and influencing factors of oral frailty in older adults: a systematic review and meta-analysis
Source: Front Public Health. 2024 Dec 13;12:1457187. doi: 10.3389/fpubh.2024.1457187 (PMC11671401; doi:10.3389/fpubh.2024.1457187)
Supplement: Supplementary file 1 [file Table_1.DOCX]

1. **Supplementary Tables**

Table S1 Search strategy on PubMed..

| **Search number** | **Query** | **Results** |
| --- | --- | --- |
| #1 | "Aged"[MeSH Terms] | [3,521,943](https://pubmed.ncbi.nlm.nih.gov/?term=) |
| #2 | "older people"[Title/Abstract] OR "older adult*"[Title/Abstract] OR "elderly"[Title/Abstract] | [453,337](https://pubmed.ncbi.nlm.nih.gov/?term=(('older+people'%5bTitle/Abstract%5d)+OR+('older+adult*'%5bTitle/Abstract%5d))+OR+(elderly%5bTitle/Abstract%5d)&sort=date) |
| #3 | #1 OR #2 | [3,643,603](https://pubmed.ncbi.nlm.nih.gov/?term=() |
| #4 | "oral frailty"[Title/Abstract] OR "oral frail"[Title/Abstract] | 135 |
| #5 | "impact*"[Title/Abstract] OR "cause*"[Title/Abstract] OR "reason*"[Title/Abstract] OR "association"[Title/Abstract] OR "relationship"[Title/Abstract] OR "effect*"[Title/Abstract] | [13,577,903](https://pubmed.ncbi.nlm.nih.gov/?term=(((((impact*%5bTitle/Abstract%5d)+OR+(cause*%5bTitle/Abstract%5d))+OR+(reason*%5bTitle/Abstract%5d))+OR+(association%5bTitle/Abstract%5d))+OR+(relationship%5bTitle/Abstract%5d))+OR+(effect*%5bTitle/Abstract%5d)&sort=date) |
| #6 | #3 AND #4 AND #5 | 81 |

Table S2 Literature quality assessment of cross-sectional studies(n=16)

| Author,year | Items | | | | | | | | | | | Overall score | Quality assessment |
| --- | --- | --- | --- | --- | --- | --- | --- | --- | --- | --- | --- | --- | --- |
|  | (1) | (2) | (3) | (4) | (5) | (6) | (7) | (8) | (9) | (10) | （11） |  |  |
| Sanae Hironaka et al., 2020 | 1 | 1 | 1 | 1 | 0 | 1 | 1 | 0 | 1 | 1 | 1 | 9 | High |
| Ryo Komatsu et al., 2021 | 1 | 1 | 1 | 1 | 0 | 0 | 1 | 0 | 1 | 1 | 0 | 7 | Moderate |
| Yuki Ohara et al., 2020 | 1 | 1 | 1 | 1 | 1 | 0 | 1 | 0 | 1 | 1 | 0 | 8 | High |
| Tomoki Tanaka et al., 2021 | 1 | 1 | 1 | 1 | 1 | 0 | 1 | 1 | 0 | 1 | 1 | 9 | High |
| Kaija Hiltunen et al., 2021 | 1 | 1 | 1 | 1 | 0 | 0 | 1 | 1 | 0 | 1 | 0 | 7 | Moderate |
| Misa Nishimoto et al., 2023 | 1 | 1 | 1 | 1 | 0 | 0 | 1 | 0 | 1 | 1 | 0 | 7 | Moderate |
| Tang et al., 2023 | 1 | 1 | 1 | 1 | 0 | 1 | 1 | 0 | 1 | 1 | 0 | 8 | High |
| Wang et al., 2023 | 1 | 1 | 1 | 1 | 1 | 0 | 1 | 0 | 0 | 1 | 0 | 7 | Moderate |
| Tu et al., 2023 | 1 | 1 | 1 | 1 | 0 | 0 | 1 | 0 | 1 | 1 | 0 | 7 | Moderate |
| Izutsu et al., 2023 | 1 | 1 | 1 | 1 | 1 | 1 | 0 | 1 | 0 | 1 | 0 | 8 | High |
| Jiao et al., 2023 | 1 | 1 | 1 | 0 | 0 | 0 | 1 | 0 | 1 | 1 | 0 | 6 | Moderate |

Table S2 (continued) Literature quality assessment of cross-sectional studies(n=16)

| Author,year | Items | | | | | | | | | | | | | | Overall score | Quality assessme |
| --- | --- | --- | --- | --- | --- | --- | --- | --- | --- | --- | --- | --- | --- | --- | --- | --- |
|  | (1) | | (2) | (3) | | (4) | | (5) | (6) | (7) | (8) | (9) | (10) | (11) |  |  |
| Iwasaki, Watanabe, et al., 2021 | 1 | 1 | | | 1 | | 1 | 1 | 0 | 1 | 1 | 0 | 1 | 0 | 8 | High |
| Wu et al., 2024 | 1 | 1 | | | 1 | | 0 | 1 | 0 | 1 | 1 | 0 | 1 | 0 | 7 | Moderate |
| Daiki Watanabe et al.,2024 | 1 | 1 | | | 1 | | 1 | 1 | 0 | 1 | 0 | 1 | 1 | 0 | 8 | High |
| Yang et al.,2024 | 1 | 1 | | | 1 | | 0 | 1 | 1 | 1 | 0 | 0 | 1 | 0 | 7 | Moderate |
| Eri Arai et al.,2024 | 1 | 1 | | | 1 | | 0 | 1 | 1 | 1 | 0 | 0 | 1 | 1 | 8 | High |

Note:Items scored: 0: no or unclear,1: yes,quality assessment: low(score 0-3), moderate(score 4-7),or high(score 8-11). (1)Define the source of information(survey, record review); (2)List inclusion and exclusion criteria for exposed and unexposed subjects (cases and controls) or refer to previous publications; (3)Indicate time period used for identifying patients; (4)Indicate whether or not subjects were consecutive if not population-based; (5)Indicate if evaluators of subjective components of study were masked to other aspects of the status of the participants; (6)Describe any assessments undertaken for quality assurance purposes (e.g., test/retest of primary outcome measurements); (7)Describe how confounding was assessed and/or controlled; (8)Describe how confounding was assessed and/or controlled; (9)lf applicable, explain how missing data were handled in the analysis; (10)Summarize patient response rates and completeness of data collection; (11)Clarify what follow-up, if any, was expected and the percentage of patients for which incomplete data or follow-up was obtained.

Table S3 Literature quality assessment of cohort studies(n=1)

| Author,year | **Selection** | | | | **Comparability** | Outcome | | | Overall score | Quality assessment |
| --- | --- | --- | --- | --- | --- | --- | --- | --- | --- | --- |
|  | (1) | (2) | (3) | (4) | (5) | (6) | (7) | (8) |  |  |
| Tanaka et al., 2023 | 1 | 1 | 1 | 0 | 1 | 1 | 1 | 1 | 7 | High |

Note: A study can be awarded a maximum of 1 point for each numbered item within the Selection and Outcome categories. A maximum of 2 points can be given for Comparability. quality assessment: low(score 0-3), moderate(score 4-6),or high(score 7-9). (1)Representativeness of the exposed cohort; (2)Selection of the non exposed cohort; (3)Ascertainment of exposure; (4)Demonstration that outcome of interest was not present at start of study; (5)Comparability of cohorts on the basis of the design or analysis; (6)Assessment of outcome; (7)Was follow-up long enough for outcomes to occur; (8)Adequacy of follow up of cohorts.
