# Supplementary figures and images for: Prevalence and influencing factors of oral frailty in older adults: a systematic review and meta-analysis
Source: Front Public Health. 2024 Dec 13;12:1457187. doi: 10.3389/fpubh.2024.1457187 (PMC11671401; doi:10.3389/fpubh.2024.1457187)

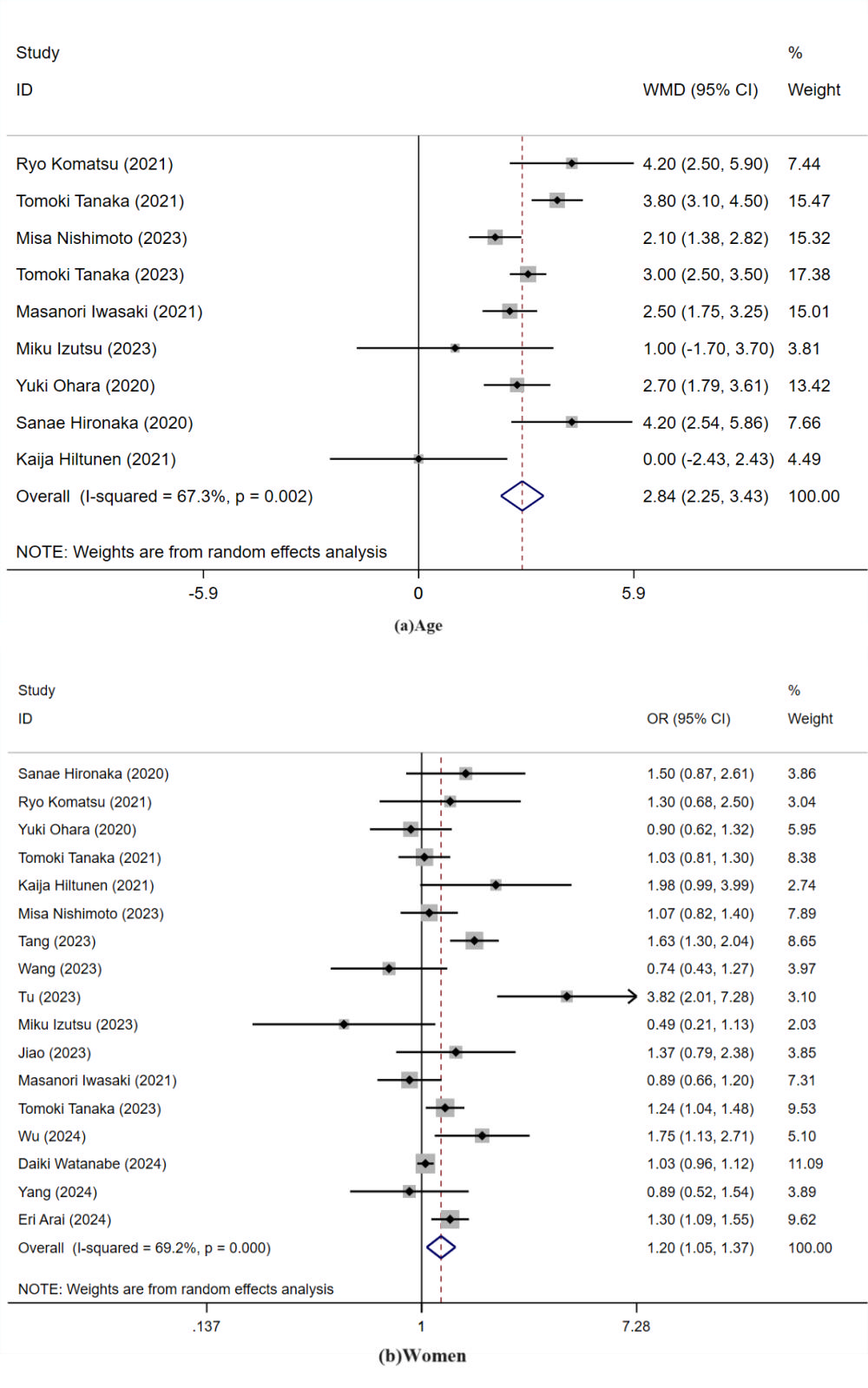

Supplement: Supplementary file 2 [file Image_1.TIFF]

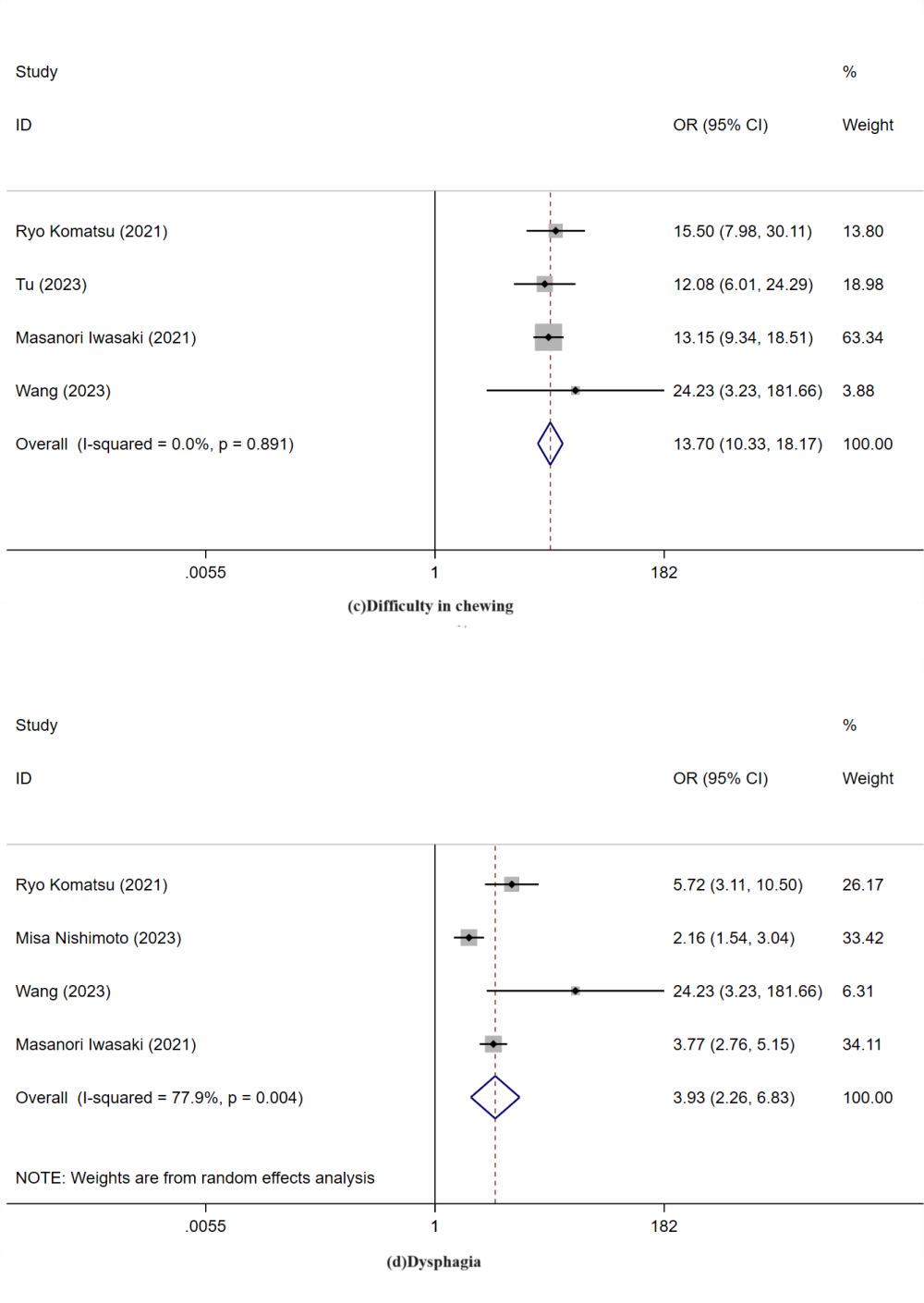

Supplement: Supplementary file 3 [file Image_2.TIFF]

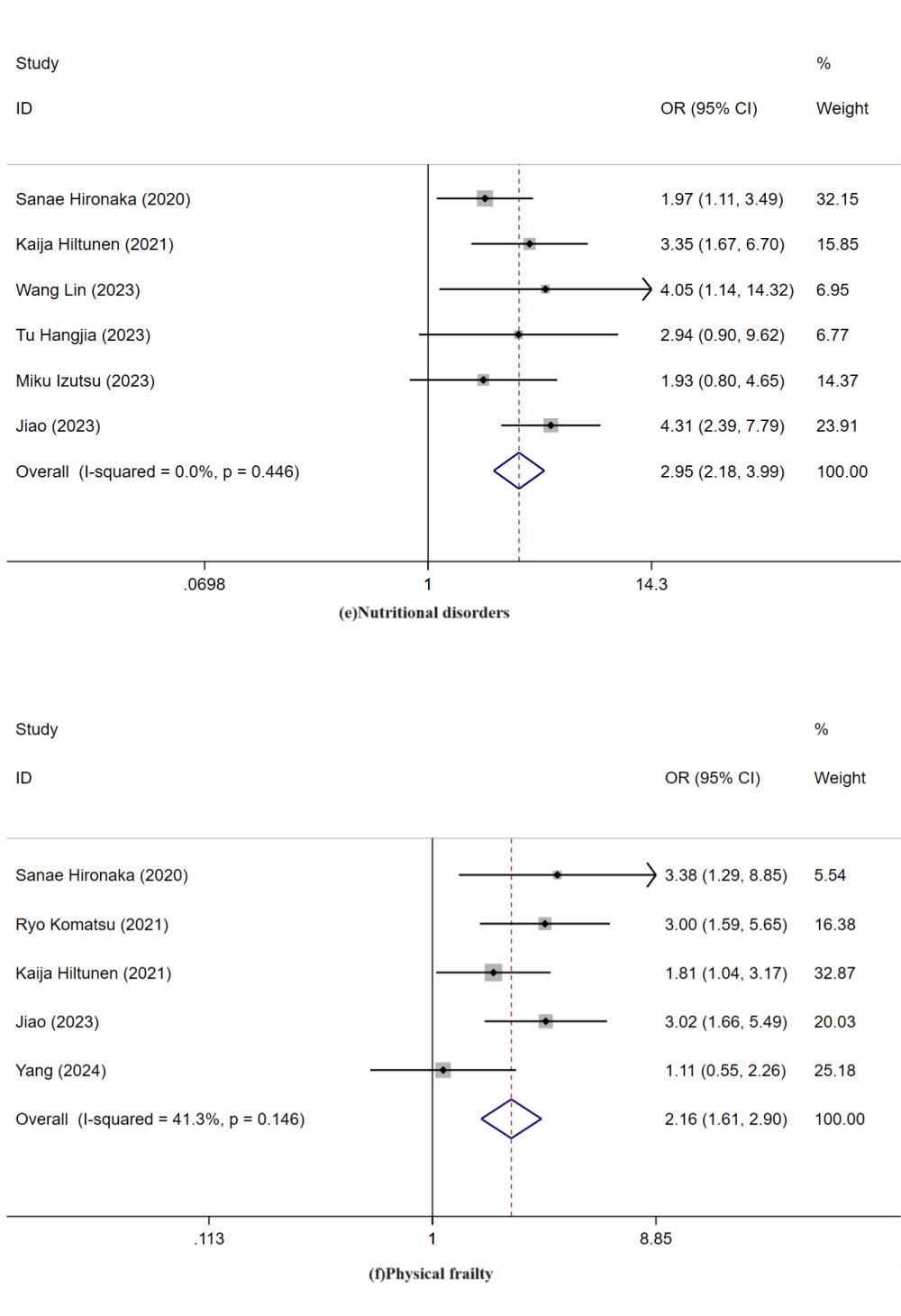

Supplement: Supplementary file 4 [file Image_3.TIFF]

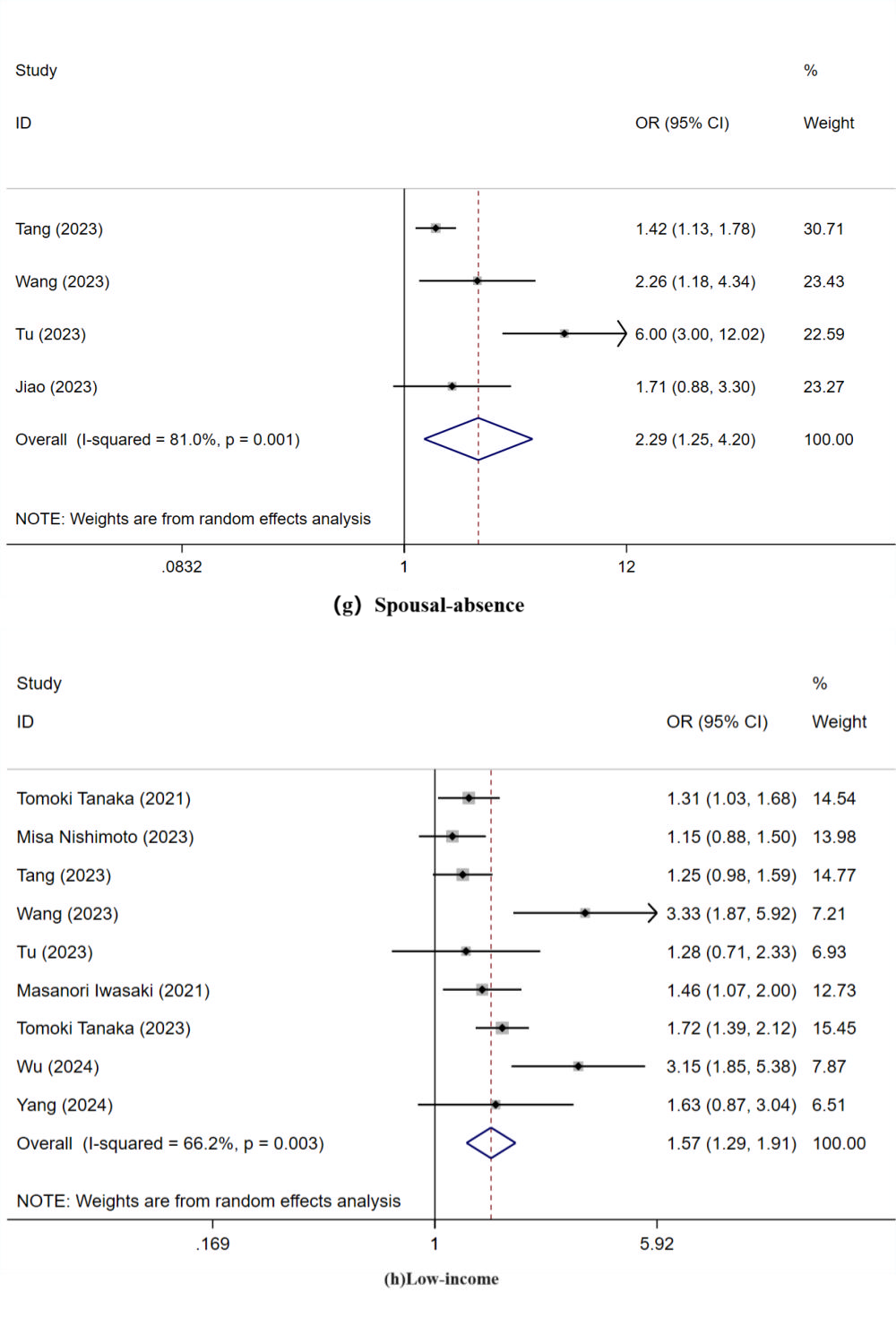

Supplement: Supplementary file 5 [file Image_4.TIFF]

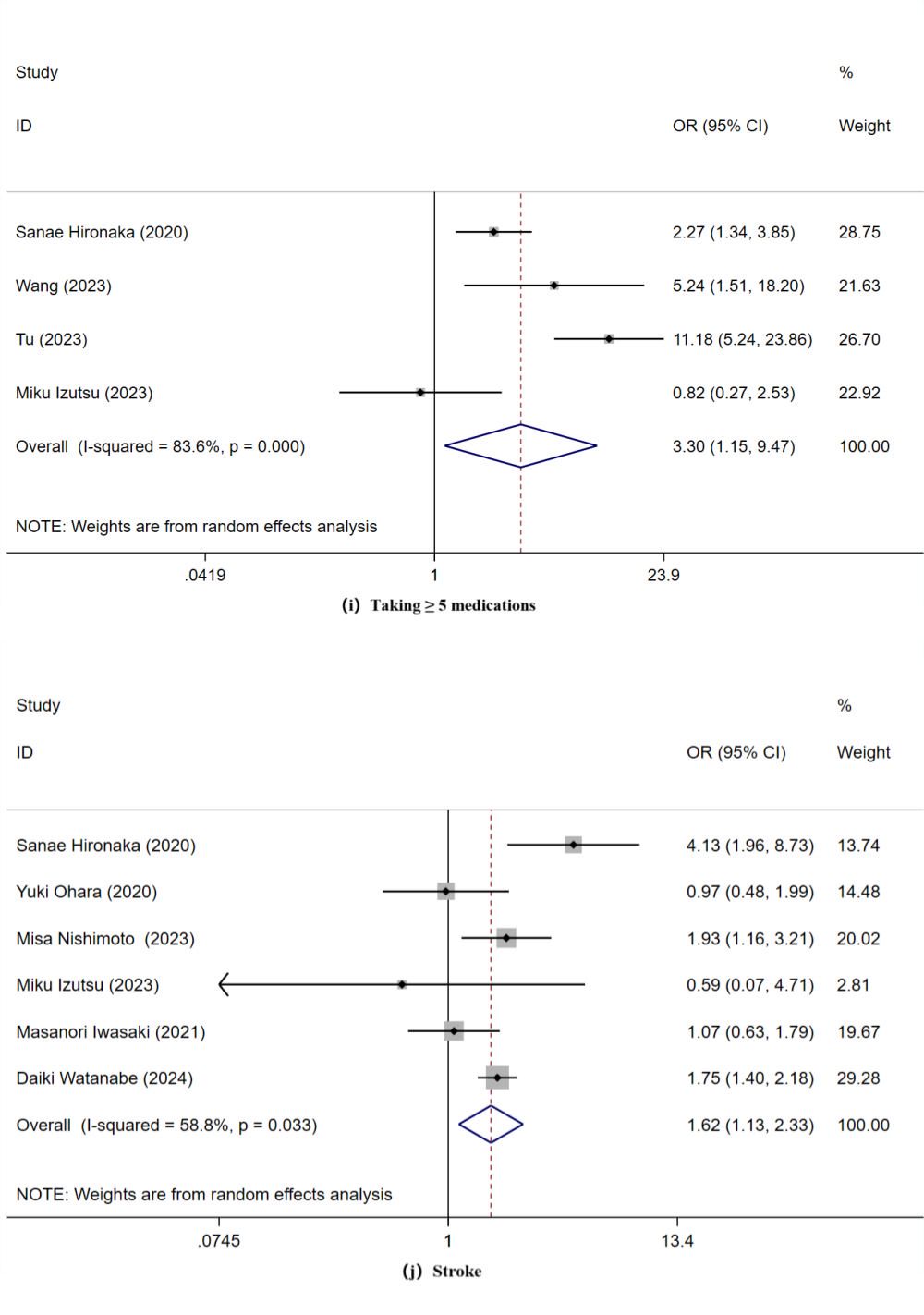

Supplement: Supplementary file 6 [file Image_5.TIFF]

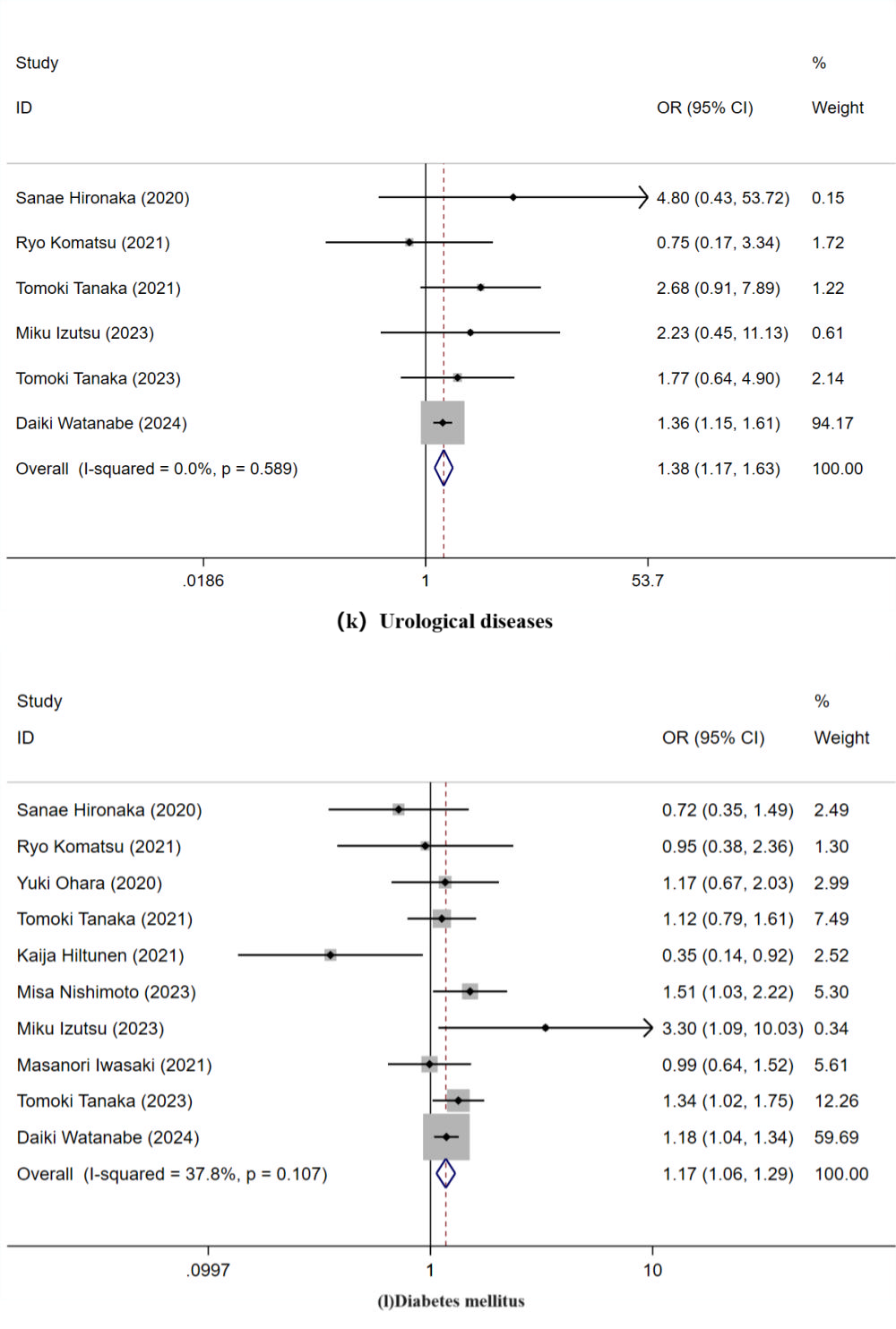

Supplement: Supplementary file 7 [file Image_6.TIFF]

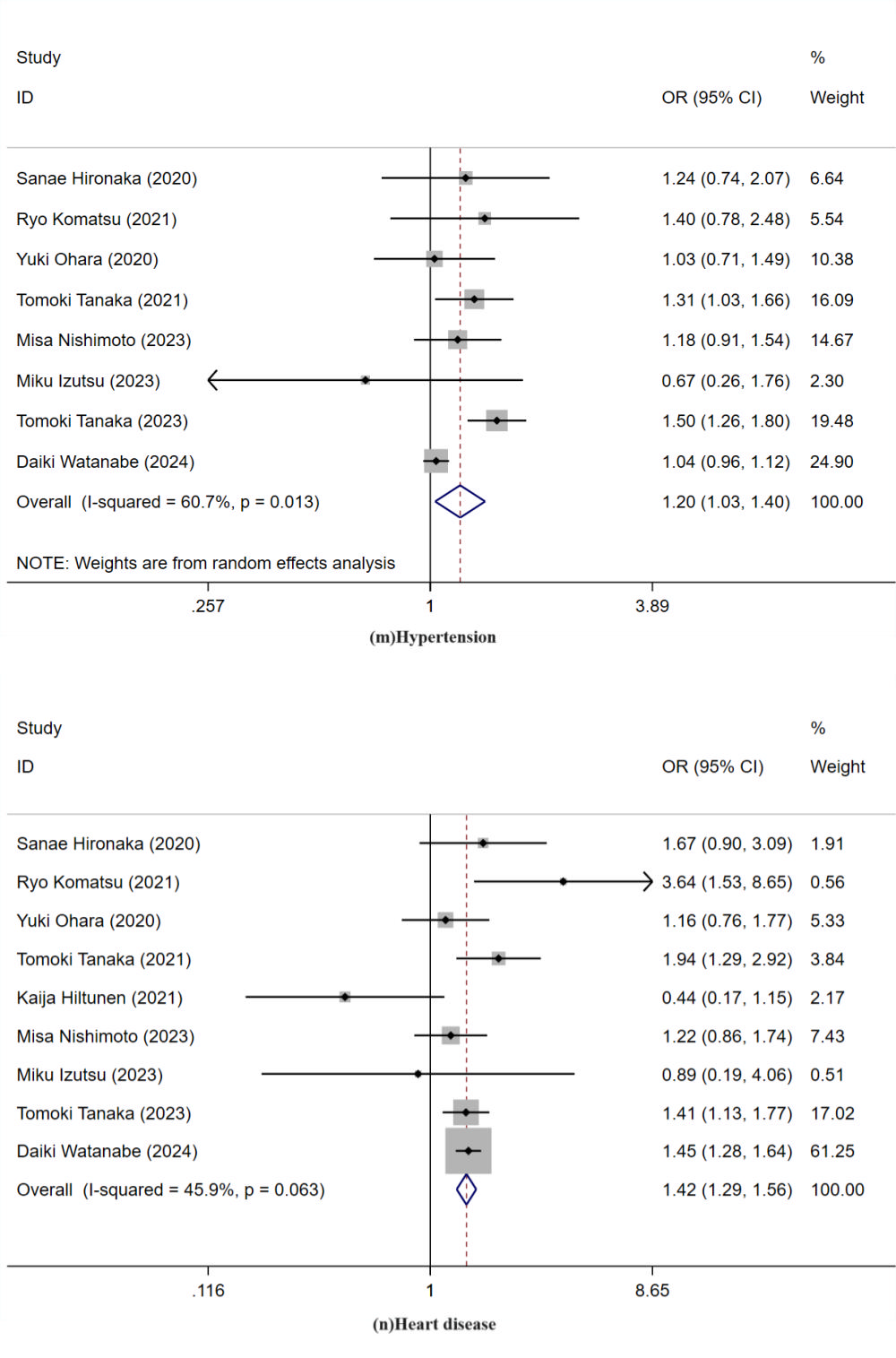

Supplement: Supplementary file 8 [file Image_7.TIFF]

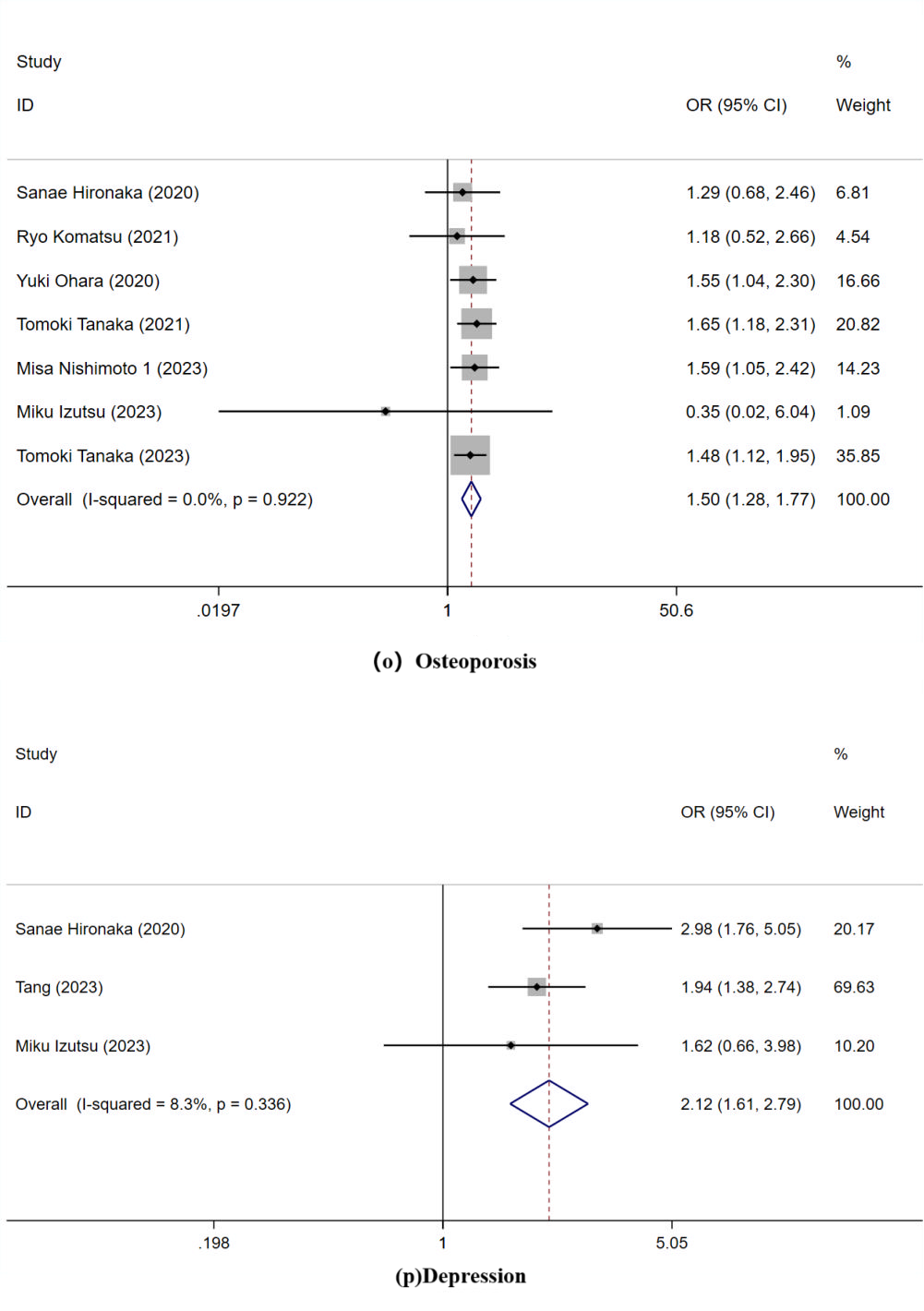

Supplement: Supplementary file 9 [file Image_8.TIFF]

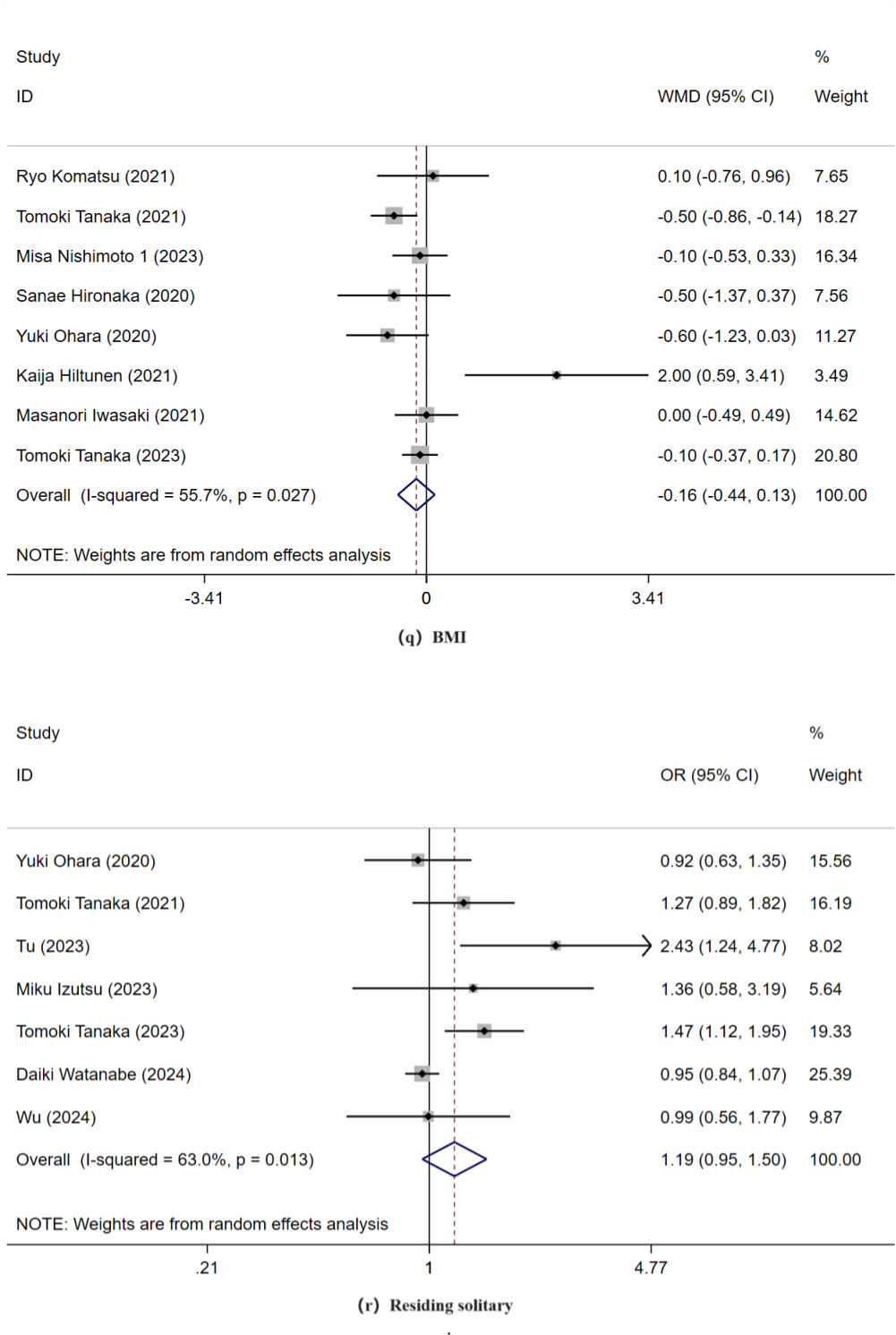

Supplement: Supplementary file 10 [file Image_9.TIFF]

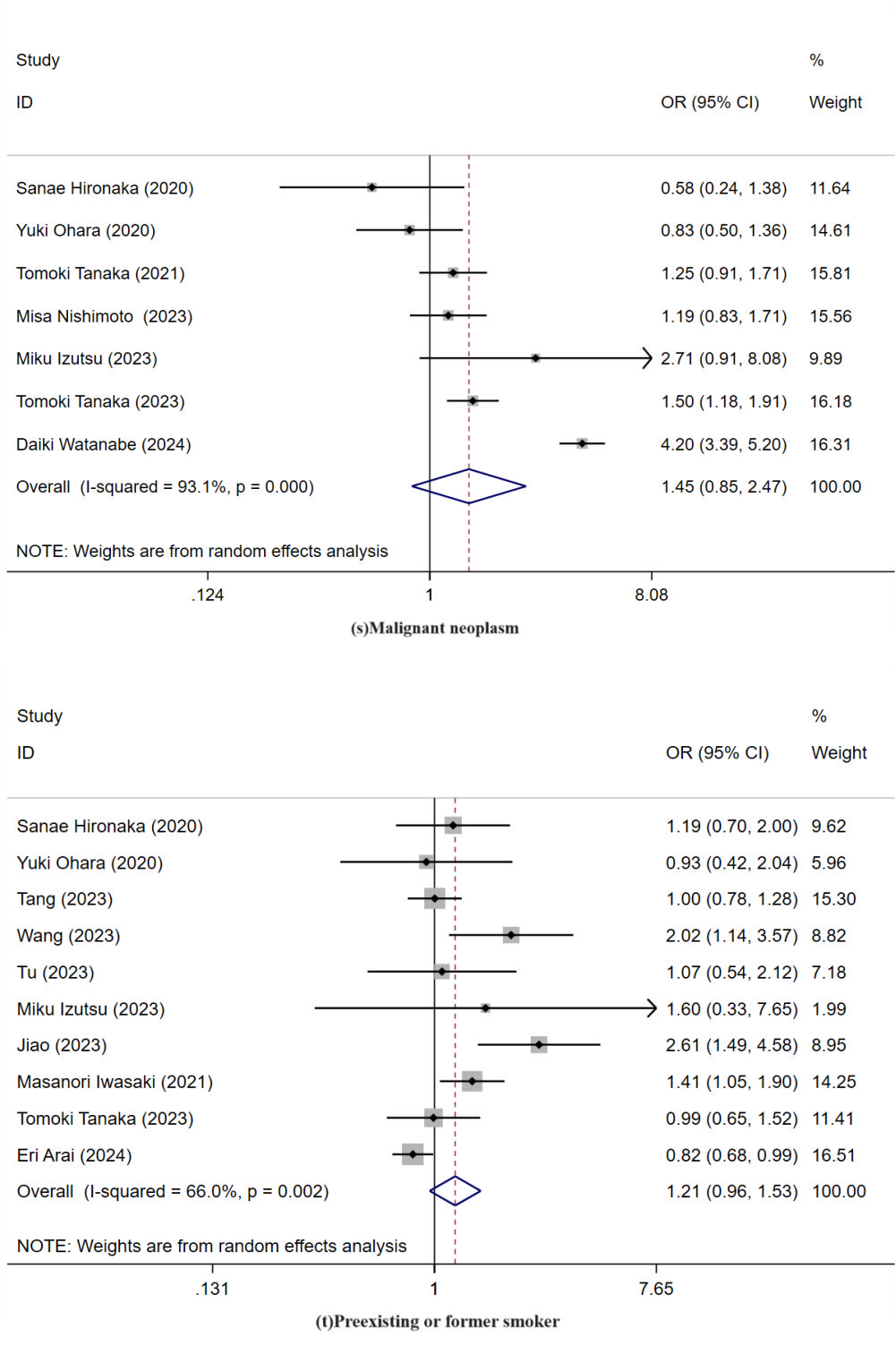

Supplement: Supplementary file 11 [file Image_10.TIFF]

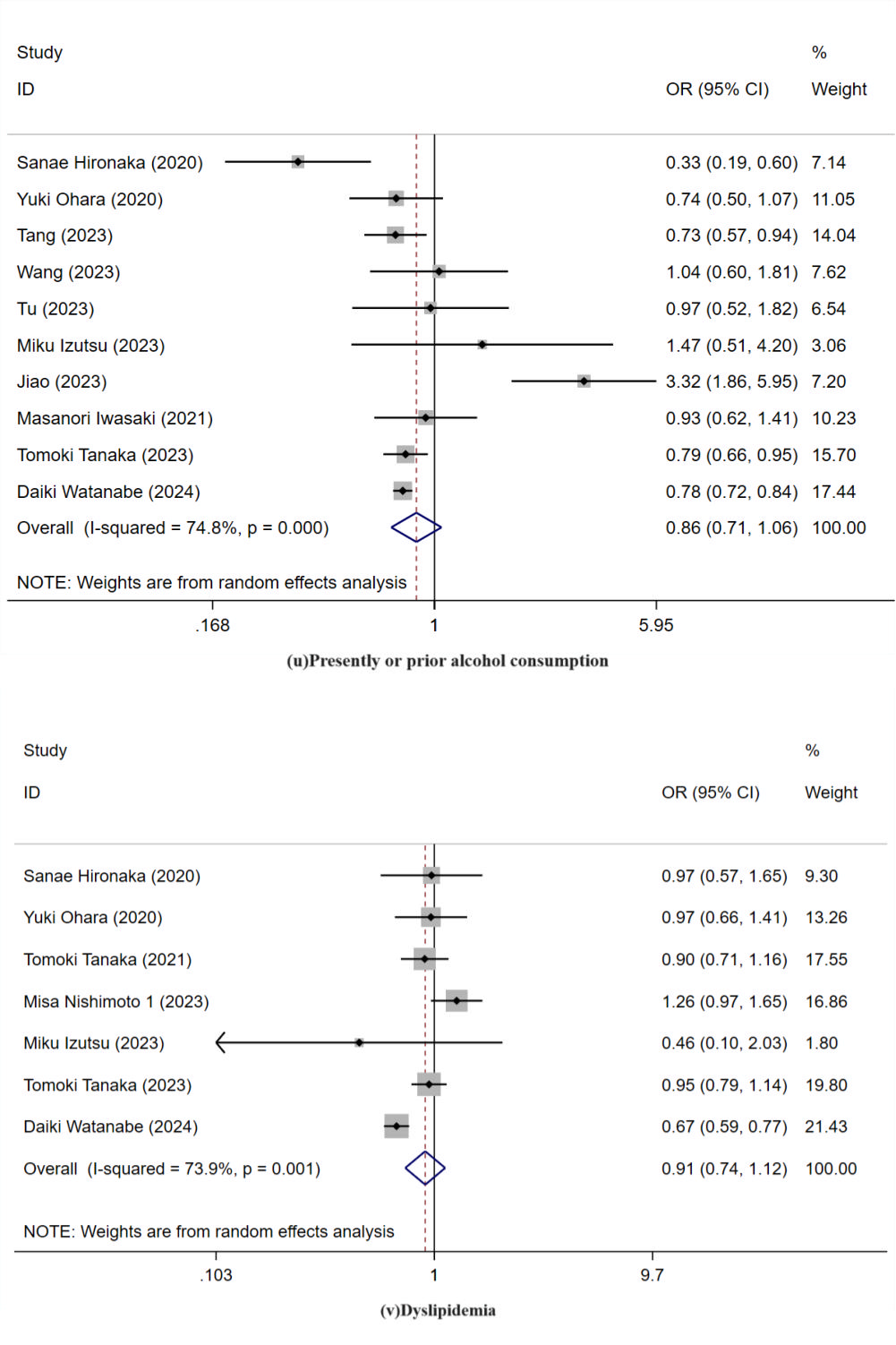

Supplement: Supplementary file 12 [file Image_11.TIFF]
